# The current status of the *Lutzomyia longipalpis* (Diptera: Psychodidae: Phlebotominae) species complex

**DOI:** 10.1590/0074-02760160463

**Published:** 2017-03

**Authors:** Nataly A Souza, Reginaldo P Brazil, Alejandra S Araki

**Affiliations:** 1 Fundação Oswaldo Cruz-Fiocruz, Instituto Oswaldo Cruz, Laboratório Interdisciplinar de Vigilância Entomológica em Diptera e Hemiptera, Rio de Janeiro, RJ, Brasil; 2 Fundação Oswaldo Cruz-Fiocruz, Instituto Oswaldo Cruz, Laboratório de Doenças Parasitárias, Rio de Janeiro, RJ, Brasil; 3 Fundação Oswaldo Cruz-Fiocruz, Instituto Oswaldo Cruz, Laboratório de Biologia Molecular de Insetos, Rio de Janeiro, RJ, Brasil

**Keywords:** Lutzomyia longipalpis, sandfly, speciation, species complex

## Abstract

*Lutzomyia longipalpis s.l.* is a complex of sibling species and is the principal vector of American visceral leishmaniasis. The present review summarises the diversity of efforts that have been undertaken to elucidate the number of unnamed species in this species complex and the phylogenetic relationships among them. A wide variety of evidence, including chemical, behavioral and molecular traits, suggests very recent speciation events and complex population structure in this group. Although significant advances have been achieved to date, differential vector capacity and the correlation between structure of parasite and vector populations have yet to be elucidated. Furthermore, increased knowledge about recent epidemiological changes, such as urbanisation, is essential for pursuing effective strategies for sandfly control in the New World.

*Historical background* - The oldest taxon in the family Psychodidae Newman (1834) is *Bibio papatasi* Scopoli 1786. Later, Rondani and Berté created the genus *Flebotomus* Rondani 1840 *,* which was subsequently modified by Agassiz (1846) to become *Phlebotomus* and ratified by the International Commission on Zoological Nomenclature in 1950 (Hemming 1958). Coquillet (1907) described the first sandflies from the Americas with *F. vexator*, from the state of Maryland, United States of America, and *F. cruciatus* from Alta Vera Paz, Guatemala. In Brazil, Lutz and Neiva (1912) described three species, among them males and females of *P. longipalpis* from the farm Ouro Fino, near Benjamin Constant (Minas Gerais state - MG) and “Mata da Saúde”, near the city of São Paulo (São Paulo state - SP). França (1920) created the subgenus *Lutzia*, and four years later replaced it with *Lutzomyia*, in which he included *P. longipalpis*. In that year, Nuñez-Tovar (1924) described the male of *P. otamae* from Carabobo state, Venezuela, and nearly two years later Dyar and Nuñez-Tovar (1926/27) placed that species name in the synonymy of *P. longipalpis.* In Mexico*,* Galliard (1934) described the female of *P. almazani* from Yucatan state, which was subsequently considered a synonym of *P. longipalpis* by Fairchild and Hertig (1958) *.* Four genera were recognised in the subfamily Phlebotominae by Theodor (1948): *Phlebotomus* and *Sergentomyia* in the Old World and *Lutzomyia* and *Brumptomyia* in the New World. Posteriorly, several proposals for revision were published with the objective of classifying and grouping the sandflies of the New World (Galati 2003). According to Barretto (1962), the American species of the subfamily Phlebotominae included the genera *Warileya*, *Brumptomyia* and *Lutzomyia*, the latter divided into fifteen subgenera, among them *Lutzomyia*. Young and Duncan (1994), reviewed the genus *Lutzomyia*, where they maintained the genus, but created the subgenera *Coromyia*, *Psathyromyia* and *Sciopemyia*. The following year, a classification of the American species with phylogenetic approach was proposed, grouping and regrouping several species, however, the genus status of *Lutzomyia* is maintained, of which *Lutzomyia longipalpis* is included (Galati 1995, 2003). Due to its widespread distribution, early doubts arose about *Lu. longipalpis* Lutz and Neiva (1912) being a single species.

*Lu. longipalpis species complex* - The first evidence of morphological differences between populations of *Lu. longipalpis s.l.* was recorded by Mangabeira Filho (1969) studying Brazilian sandflies. Male sandflies collected in Pará state (PA) (North region of Brazil) had one pair of pale tergal spots on abdominal tergite IV (the one-spot phenotype named ‘1S’), while the males from Ceará state (CE) (Northeast region of Brazil) had two pairs of spots (the two-spot phenotype named ‘2S’), one on tergite IV and another on tergite III. Additionally, Mangabeira observed ecological differences between the sandflies of these two collection sites and suggested the existence of different species or varieties. Later, the observation of high-frequencies of intermediate phenotypes (a pair of pale spots with a smaller spot on the tergite III) indicated that this character is actually an intraspecific polymorphism (Ward et al. 1988) (Fig. 1).

**Fig. 1:**
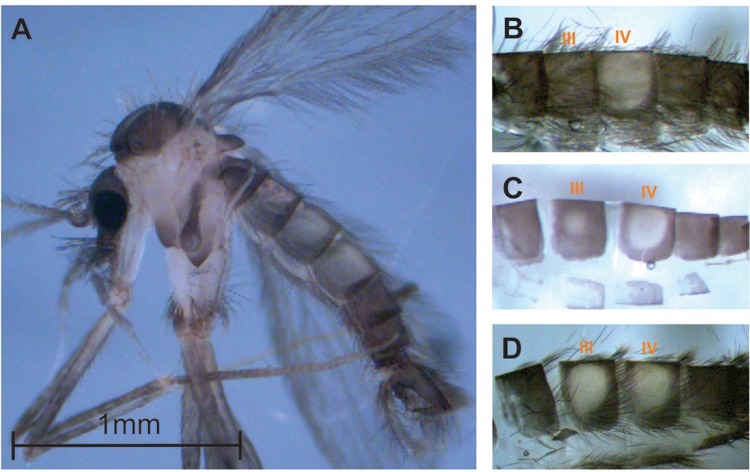
: male of *Lutzomyia longipalpis* showing morphological variation in the tergal pale spot pattern. (A) General overview of the body; (B) one-spot phenotype; (C) intermediate phenotype; (D) two-spot phenotype. III and IV: third and fourth abdominal tergites, respectively. Bar = 1 mm.

Fourteen years after the first recognition of the spot phenotypes, Ward et al. (1983) obtained concrete evidence to support Mangabeira’s hypothesis after carrying out crossing experiments with Brazilian populations of *Lu. longipalpis s.l.* from Marajó Island (PA / phenotype 1S), Sobral (CE / 1S and 2S phenotypes) Morada Nova (CE / phenotype 2S) and Lapinha Cave (MG / phenotype 1S). The failure of insemination between allopatric populations with similar tergal spot patterns and between sympatric populations with two dissimilar phenotypes (1S and 2S) strongly indicated the existence of additional forms in an apparent species complex.

Interest in the taxonomic status of *Lu. longipalpis s.l.* increased in the subsequent years (reviewed by Uribe 1999, Bauzer et al. 2007). Analyses involving populations from several countries of Latin America strongly supported the species complex hypothesis. Different approaches were used, both alone and integrated, and all pointed to the existence of a *Lu. longipalpis* species complex. Such analyses included isoenzyme electrophoresis (Morrison et al. 1995, Lanzaro et al. 1998, Mutebi et al. 2002), assessment of the genetic polymorphism of vasodilator peptide maxadilam DNA (Warburg et al. 1994, Lanzaro et al. 1999) and mRNA (Yin et al. 2000), cytogenetics (Yin et al. 1999), measurement of nucleotide variation in the *NADH dehydrogenase subunit* 4 - *ND4* (Soto et al. 2001) and *cytochrome c oxidase* I - *COI* (Arrivillaga et al. 2002) mitochondrial genes. Variation at microsatellite loci was found to be related to male pheromone type (Watts et al. 2005), and isoenzyme electrophoresis was combined with crossing experiments (Lanzaro et al. 1993), wing morphometry (Dujardin et al. 1997) and single strand conformation polymorphism analysis of *COI*, 12S and 16S rRNA genes (Arrivillaga et al. 2003), and all supported the species complex hypothesis. Given all of this evidence, there was no more doubting the existence of a *Lu. longipalpis* species complex that is distributed over a broad area spanning the Neotropic region (Table).

**TABLE:** Summary of studies of the *Lutzomyia longipalpis* species complex

Species	CC	Localities	Morphology	Crossing	Pheromone	Song/ behavior	Molecular markers					Citogenetic	Maxadilan

							Isoenzyme	Mitochondrial	Microsatellite	Nuclear	RAPD		
*Lu. longipalpis*	AR	Posadas (Misiones)			9MGB^50^					*per* ^50^			
	BO	Apa Apa, Guayabal and Imanaco (La Paz), Toro Toro (Potosi)	[^13^]				[^13^]						
		Yungas (La Paz)					[^5^]						
	BR	Abaetetuba (Pará-PA)					[^10^]						
		Adamantina, Araçatuba, Bauru, Dracena, Jales, Lourdes, Marília, Oswaldo Cruz, Presidente Prudente, Promissão and Salmourão (São Paulo-SP)			9MGB^56^								
		Afonso Cláudio (Espírito Santo-ES), Aracajú (Sergipe-SE), Barcarena and Cametá (PA), Ipanema and Nova Porteirinha (Minas Gerais-MG), Passira (Pernambuco-PE)			Cemb-1^60^	B^58^							
		Aquidauana, Bonito and Miranda (Mato Grosso do Sul-MS)	[^55^]						[^53^]				
		Bacabal (Maranhão-MA)					[^19, 31, 33^]	*COI*, 12S and 16S^33^		*per* ^49^			
		Fortaleza and Itapipoca (Ceará-CE)					[^19, 31, 33^]	*COI*, 12S and 16S^33^					
		Barra de Guaratiba (Rio de Janeiro-RJ)			9MGB^45^					*per* ^46^			
		Baturite (CE)					[^19, 31, 33^]	*COI* ^30^; *COI*, 12S and 16S^33^		*per* ^49^			
		Belo Horizonte (MG), Niteroi, Rio Bonito and Saquarema (RJ)			9MGB^60^								
		Bodocó (PE), Caririaçú (CE)								1S/2S*: per* ^57^			
		Cáceres (Mato Grosso-MT)						*COI* ^59^					
		Calumbi (PE), João Pessoa and Patos (PA)						*cyt b* ^42^			[^40^]		
		Camaçari (Bahia-BA)									[^40^]		
		Camará (PA)			Cemb-1^60^	B^58^	[^19, 31, 33^]	*COI*, 12S and 16S^33^					
		Campo Grande (MS)	1S, 2S: [^55^]		9MGB^56^				[^53^]				
		Canindé (CE), São José de Ribamar (MA)	[^22^]				[^22^]						
		Cavunge and Jequié (BA)			3MαH^60^	P1^58^							
		Aguas da Prata, Campinas, Espírito Santo do Pinhal, Indaiatuba, Salto, Socorro, Votorantin (SP)			Cemb-1^56^								
		Estrela de Alagoas (Alagoas-AL)	1S, 2S: [^55^]		1S: Cemb-1^39^ 2S: Cemb-1^39^	1S: P5^46^ 2S: B^46^			[^53^]	1S, 2S: *per* ^46^, *para* ^52^			
		Feira de Santana, Juazeiro and Monte Santo (BA)						*cyt b* ^34, 42^					
		Itamaracá (PE)			Cemb-1^60^	B^58^		*cyt b* ^34, 42^			[^40^]		
		Jacobina (BA)		[^6, 41^]	3MαH^11c^	P1^27^ , [^45^]	[^16, 19, 31, 33^]	*ND4* ^26^, *COI* ^30^, *COI*, 12S and 16S^33^	[^38^]	*per* ^28a^, *cac* ^25,35^, *para* ^52^		[^17^]	[^21^]
		Jaíba (MG)			1S: Cemb-2^36^ 2S: Cemb-1^36^	1S: P4^46^ 2S: B^46^				1S/2S: *per* ^46,^ *para* ^52^			
		Lapinha (MG)	[^22^]	[^2, 6, 8, 41^]	9MGB^3, 11a, 39^	P2^27^ , [^45^]	[^8, 16, 15, 10, 19, 22 , 31, 33^]	*ND4* ^26^, *COI* ^30^, *COI*, 12S and 16S^33^	[^38^]	*per* ^28a^, *cac* ^25,35^, *para* ^52^, #^54^		[^17^]	[^9, 18, 21^]
		Lassance (MG), Pirenópolis (Goiás-GO)			9MGB^60^	P4^58^							
		Maceió (AL)			Cemb-1^60^								
		Marajó (PA)		[^2, 6^]	Cemb-1^4^	B^37^	[^16^]		[^38^]	*per* ^37^ *, para* ^52^			[^18^]
		Mesquita (RJ)				Mix^46^				*per* ^46^			
		Montes Claros (MG)			9MGB^39^		[^19, 31, 33^]	*COI*, 12S and 16S^33^					
		Morada Nova (CE)		[^2,6^]	Cemb-1^3^								
		Natal (RN)	[^22^]	[^41^]	Cemb-1^38^	B^27^, [^45^]	[^16, 22^]	*cyt b* ^34, 42^	[^38^]	*per* ^28a^, *cac* ^35^, *para* ^52^			
		Pacaraima Montains (Roraima-RO)					[^33^]	*COI* ^30^, *COI*, 12S and 16S^33^		*per* ^49^			
		Palmas (Tocantins-TO)				1S: P4^58^ 2S: B^58^							
		Pancas (ES)			Cemb-1^46^	B^46^	[^16^]	*cyt b* ^34, 42^, *COI* ^59^		*per* ^46^, *cac* ^35^, *para* ^52^, #^54^			
		Porto Nacional (TO)			9MGB^51^ Cemb-1^51^								
		Russas & Icó (CE)	[^1^]										
		Salvaterra (PA)	[^22^]				[^19, 22, 31^]	*COI* ^30^					
		San Pedro (SP)			9MGB + Cemb-1^56^								
		Santarém (PA)			Cemb-1^4, 39^		[^16, 19, 31, 33^]	*COI* ^30^, *COI*, 12S and 16S^33^					
		São Luiz (MA)		[^6^]	9MGB^60^		[^16^]			*per* ^49^	[^40^]		
		Sobral (CE)		[^2, 6, 41^]	1S: 9MGB^4, 39^ 2S: Cemb-1^6, 39^	1S: P3^37^, [^43, 45^] 2S: B^37^, [^43, 45^]	[^12, 19, 31, 33^]	*ND4* ^26^, *COI*, 12S and 16S rRNA^33^	[^32, 38^]	1S/2S: *per* ^28b,49,57^, *cac* ^35^, *para* ^44^, #^54^			
		Sol da Costa (AL)			Cemb-1^39^								
		Sorocaba (SP)			Cemb-1^56^								
		Teresina (Piauí-PI)			9MGB^46^	P3^46^				*per* ^46,^ *para* ^52^	[^40^]		
		Três Lagoas (MS)	1S, 2S: [^55^]		9MGB^60^				[^53^]				
	CO	Bucaramanga (Santander)					[^31, 33^]	*COI* ^30^, *COI*, 12S and 16S^33^					
		Duranía (Santander)					[^15, 31, 33^]	*COI*, 12S and 16S^33^					[^18^]
		El Callejón (Huila)					[^10, 16^]	*ND4* ^26^				[^17^]	[^18, 21^]
		Girón (Santander)						*ND4* ^26^					
		L’Aguila (Tolima)			9MGB^7^								
		Melgar (Tolima)		[^8^]			[^8, 10, 12, 15^]						[^9^]
		Neiva (Huila)					[^15, 31, 33^]	*ND4* ^26^, *COI* ^30^, *COI*, 12S and 16S^33^		*per* ^49^			[^18^]
		Palo Gordo (Santander)					[^15, 31, 33^]	*COI*, 12S and 16S^33^					
	CR	Brasilito (Guanacaste)					[^14, 31, 33^]	*COI*, 12S and 16S^33^		*per* ^49^			[^18, 21^]
		Liberia (Guanacaste)		[^8^]	9MGB^11b,^		[^8, 14, 20, 31, 33^]	*ND4* ^26^, *COI* ^30^, *COI*, 12S and 16S^33^		*per* ^49^		[^17^]	[^9, 18, 21^]
		Northern					[^10^]						
	GT	Tulumajillo (El Progreso)						*ND4* ^26^					
	HN	Isla El Tigre, Los Guatales, Rancho Grande, San Francisco del Coray					[^14, 31, 33^]	*ND4* ^26^, *COI*, 12S and 16S^33^					
		Orocuina, Pavana and San Juan Batista (Choluteca)					[^14, 31, 33^]	*COI*, 12S and 16S^33^					
		Tololar (Choluteca)			9MGB^11b^								
	NI	Cinco Pinos, Somotillo	[^13^]										
		Las Huertas, Pochomil					[^14, 31, 33^]	*COI*, 12S and 16S^33^					
	PA	Vila Elisa (Asunción)			9MGB^47^								
	VE	Altagracia (Guarico)					[^20^]		[^38^]				
		El Pao (Cojedes)	[^24^]				[^23, 33^]	*COI*, 12S and 16S^33^					
		Curarigua (Lara), Trujillo	[^24^]				[^23, 33^]	*COI* ^30^, *COI*, 12S and 16S^33^		*per* ^49^			
		El Layero (Guarico)			9MGB^38^				[^38^]				
		El Paso (Lara)					[^20, 33^]	*COI*, 12S and 16S^33^					
		Guayabita (Aragua)	[^24^]		9MGB^38^		[^23^]		[^38^]				
		Las Cabreras (Nueva Esparta)							[^38^]				
		La Rinconada (Lara), Mapire (Anzoategui)					[^20^]						
*Lu. cruzi*	BR	Cáceres (MT)						*COI* ^59^					
		Corumbá (MS)	1S, 2S: [^55^]		9MGB^29^	B^48^			[^38,53^]	*per* ^48,^ *para* ^52^			
		Ladário (MS)			9MGB^38^				[^38^]				
	BO	El Carmen			9MGB^60^								
*Lu. pseudolongipalpis*	VE	El Paso (Lara)			3MαH^24^								
		La Rinconada (Lara)	[^24^]		3MαH^38^				[^38^]	*per* ^49^			

CC: country codes; AR: Argentina; BO: Bolivia; BR: Brazil; CO: Colombia, CR: Costa Rica; GT: Guatemala; HN: Honduras; NI: Nicaragua; PA: Paraguay; VE: Venezuela. References: ^1^Mangabeira Filho (1969); ^2^Ward et al. (1983); ^3^Lane et al. (1985); ^4^Phyllips et al. (1986); ^5^Bonnefoy et al. (1986); ^6^Ward et al. (1988); ^7^Hamilton and Ward (1991); ^8^Lanzaro et al. (1993); ^9^Warburg et al. (1994); ^10^Morrison et al. (1995); ^11a^Hamilton et al. (1996a); ^11b^Hamilton et al. (1996b); ^11c^Hamilton et al. (1996c); ^12^Mukhopadhyay et al. (1997); ^13^Dujardin et al. (1997); ^14^Mutebi et al. (1998); ^15^Lanzaro et al. (1998); ^16^Mukhopadhyay et al. (1998b); ^17^Yin et al. (1999); ^18^Lanzaro et al. (1999); ^19^Mutebi et al. (1999); ^20^Lampo et al. (1999); ^21^Yin et al. (2000); ^22^de Azevedo et al. (2000); ^23^Arrivillaga et al. (2000); ^24^Arrivillaga and Feliciangeli (2001); ^25^Oliveira et al. (2001); ^26^Soto et al. (2001); ^27^Souza et al. (2002); ^28a^Bauzer et al. (2002a); ^28b^Bauzer et al. (2002b); ^29^Brazil and Hamilton (2002); ^30^Arrivillaga et al. (2002); ^31^Mutebi et al. (2002); ^32^Maingon et al. (2003); ^33^Arrivillaga et al. (2003); ^34^Hodgkinson et al. (2003); ^35^Bottechia et al. (2004); ^36^Hamilton et al. (2004); ^37^Souza et al. (2004); ^38^Watts et al. (2005); ^39^Hamilton et al. (2005); ^40^Balbino et al. (2006); ^41^Souza et al. (2008); ^42^Coutinho-Abreu et al. (2008); ^43^Rivas et al. (2008); ^44^Lins et al. (2008); ^45^Souza et al. (2009); ^46^Araki et al. (2009); ^47^Brazil et al. (2009); ^48^Vigoder et al. (2010); ^49^Golczer and Arrivillaga (2010); ^50^Salomón et al. (2010); ^51^Brazil et al. (2010); ^52^Lins et al. (2012); ^53^Santos et al. (2013); ^54^Araki et al. (2013); ^55^Santos et al. (2015); ^56^Casanova et al. (2015); ^57^Lima-Costa Jr et al. (2015); ^58^Vigoder et al. (2015); ^59^Pinto et al. (2015); ^60^Spiegel et al. (2016). #: 18 additional nuclear markers analysed by Araki et al. (2013) (*CG9297, CG9769, eno, kinC, mlcc, norpA, obp19a, rpL17A, rpL36, rpS19, sesB, slh, sec22, sod2, tfIIAL, tropC, up, ζcop*).

The first evidence of the existence of the *Lu. longipalpis* species complex was obtained in Brazil, yet initial studies using populations of sandflies collected in this country resulted in conflicting findings. A group of studies, mainly using isoenzyme electrophoresis, supported the single species hypothesis (Mukhopadhyay et al. 1997, 1998a, b, Mutebi et al. 1999, de Azevedo et al. 2000, Arrivillaga et al. 2003, Hodgkinson et al. 2003, Balbino et al. 2006). However, a number of them also identified some degree of genetic structure consistent with intraspecific variation (Mukhopadhyay et al. 1997, 1998a, b, Mutebi et al. 1999, de Azevedo et al. 2000, Hodgkinson et al. 2003). Isoenzyme electrophoresis has become an informational approach for distinguishing species when comparing populations that are quite different. For example, studies with Venezuelan populations showed strong evidence for the species complex hypothesis and suggested greater genetic structuring than the Brazilian studies (Lampo et al. 1999, Arrivillaga et al. 2000). Moreover, additional evidence from morphometric characters has allowed the formal recognition in Venezuela of *Lu. pseudolongipalpis* as the first species of the *Lu. longipalpis* species complex (Arrivillaga & Feliciangeli 2001).

There are a large number of studies in Brazil that strongly support the species complex hypothesis. One of the earliest, and most conclusive, studies was the crossing experiments carried out by Ward et al. (1983), mentioned previously. The efforts of Richard Ward and collaborators in studying this species complex continued for several years. They showed the existence of reproductive isolation between Brazilian populations and an association between insemination rate and specific male pheromones (Ward et al. 1985, 1988). In addition, it became apparent that the spot phenotype could not be used to identify cryptic species in all locations. A decade later, Souza et al. (2008) carried out crosses among populations from Natal (Rio Grande do Norte state - RN), Jacobina (Bahia state - BA), Lapinha (MG) and Sobral (CE) and confirmed the association previously described by Ward et al. (1988) (Fig. 2).

**Fig. 2:**
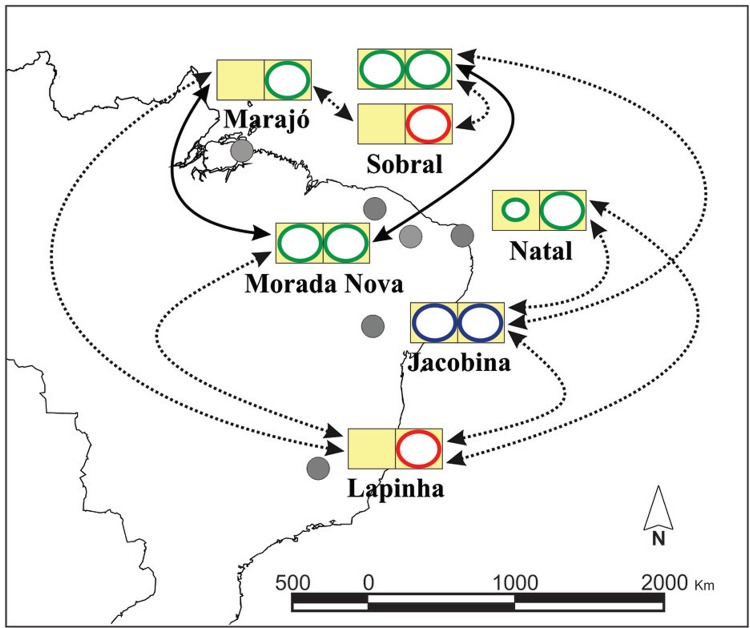
: crossing relationships between Brazilian populations of *Lutzomyia longipalpis*. Differential male pheromones: Cembrene-1 in green (Cemb-1), (*S*)-9-methyl-germacrene-B (9MGB) in red and (*1S,3S,7R*)-3-methyl-α-himachalene (3MαH) in blue. Variation in tergal spot pattern is shown with one and two circles representing the one- and two-spot phenotypes, respectively; two circles of unequal size represent the intermediate phenotype. Solid and dashed arrows indicate a normal and reduced insemination rate, respectively. Data modified from Ward et al. (1988) and Souza et al. (2008).

Analysis of the pale abdominal spots by scanning electron microscopy showed the presence of cuticular papules with central pores suggesting that they are sites of pheromone release (Lane & Ward 1984, Spiegel et al. 2002). Later, Morton and Ward (1989) demonstrated the attraction of females to tergal gland extracts, further indicating the aggregation-sex pheromone function of these compounds. From the chemical point of view, pheromones are comprised of a main and several minor components, which are responsible for attracting the female and pheromone enhancement, respectively (Hamilton et al. 1994). The analysis of different chemical compounds obtained from different populations of *Lu. longipalpis s.l.* was mainly based on the major components identified as homofarnesene (C_16_H_26_) and diterpenoids (C_20_H_32_) (Lane et al. 1985, Phyllips et al. 1986). Presently two types of homofarmasene are known, chemotype 1 or (*S*)-9-methyl-germacrene-B (9MGB) and chemotype 2 or (*1S,3S,7R*)-3-methyl-α-himachalene (3MαH); and two types of diterpenoids, the chemotype 3 or Cembrene-1 (Cemb-1) and chemotype 4 or Cembrene-2 (Cemb-2). A fifth chemotype, the chemotype 5 or 9-methyl-germacrene-B^+^ (9MGB^+^), was also identified as a mixture of compounds with a higher proportion of 9MGB (Brazil & Hamilton 2002 , Hamilton et al. 2004, 2005). A current and comprehensive review of aggregation-sex pheromones of *Lu. longipalpis s.l.* shows that 9MGB is the most predominant pheromone-type in Latin America, and is also found in *Lu. cruzi* from Brazil and Bolivia. The pheromone type 3MαH is more restricted, having only been observed in the eastern region of BA (Northeast region of Brazil) and described in *Lu. pseudolongipalpis* from La Rinconada and El Paso (Venezuela). Of the diterpenoids, Cemb-1 has been founded only in the Southeast, Midwest, Northeast and North regions of Brazil, and Cemb-2 was only detected in Jaíba, a locality in northern MG (Southeast region of Brazil) (Table) (reviewed by Spiegel et al. 2016). Pheromones are complex multifaceted signals that can have different functions, such as the recognition of individuals of the same species or recognition of a partner for mating or mate assessment (Johansson & Jones 2007, Steiger & Stökl 2014), and represent an interesting trait for studying the evolution of a species complex.

*Behavior and courtship song* - Towards the end of the 1980’s, Ward et al. (1988) observed that male and female *Lu. longipalpis s.l.* produce sounds by wing movement. This wing-flapping could be observed during aggression between males, and during courtship and mating between males and females. Moreover, auditory signaling was described for the first time in two samples, Sobral 1S and Sobral 2S, which differed in burst repetition rates and intraburst frequencies of pre-copulatory songs, and thus raised all kinds of questions about the relationships between these signals and reproductive isolation in *Lu. longipalpis s.l.* (Hoikkala & Crossley 2000, Hoikkala et al. 2000). More recently, the full sequence of pre-mating behaviors has been described (Bray & Hamilton 2007). Regarding courtship behaviors, the approach-flapping and semi-circling performed by males and the stationary-flapping of females were found to be predictors of eventual copulation. Interestingly, during copulation, females remained stationary whereas males vibrated their wings producing a species-specific song.

At the beginning of the 2000’s, Alexandre Peixoto and collaborators initiated studies of song patterns emitted during the copulations and demonstrated that this trait can identify incipient species within the *Lu. longipalpis* species complex (Souza et al. 2002, 2004). The effective insemination of females seems to depend on the patterns of these songs, and can explain the reproductive isolation observed previously by Ward et al. (1983, 1988). Males of *Lu. longipalpis s.l*. produce two different copulatory courtship songs called primary and secondary songs (Souza et al. 2002, 2004). The primary song varies and, at present, three main types have been found in the *Lu. longipalpis* species complex: Burst-type, Pulse-type and Mix-type (Souza et al. 2004, Araki et al. 2009). The Burst-type song is composed of trains with highly polycyclic pulses modulated in frequency and amplitude. The Pulse-type song is more variable and five different patterns (subtypes P1 to P5) have been identified from among Brazilian populations. Finally, the Mix-type song has a pattern that is a mixture between Burst- and Pulse-type songs, and to date has only been detected in Mesquita (Rio de Janeiro state - RJ). More recently, Vigoder et al. (2015) carried out a more geographically comprehensive analysis and corroborated the five distinct patterns of Pulse-type songs with geographical separation and no overlap among their distributions. The group of Burst-type populations had a more widespread distribution spanning the five eco-regions of Brazil. Interestingly, sympatric coexistence of the Pulse-type and Burst-type populations occur in at least four localities: Sobral, Estrela de Alagoas (Alagoas state - AL), Jaíba and Palmas (Tocantins state - TO). The recognition of male aggregation-sex pheromones by conspecific females, as mentioned previously, and cryptic female auditory choice during copulation seem to be critical for pre-zygotic reproductive isolation among sibling species of *Lu. longipalpis s.l*. (Maingon et al. 2008a, Vigoder et al. 2013).

*Molecular evidence* - The absence of diagnostic morphological characters combined with evidence obtained from other sources of data have stimulated the implementation of approaches (Table). Beginning in the early 2000’s, Alexandre Peixoto and collaborators started studying population genetics with nuclear markers in order to clarify the taxonomic status of Brazilian *Lu. longipapis s.l.*. Independently, polymorphisms of the loci *period* (*per*), *cacophony* (*cac*) and *paralytic* (*para*) were examined and found to strongly support the existence of the Brazilian species complex (Bauzer et al. 2002a, b, Bottecchia et al. 2004, Lins et al. 2008). In *Drosophila*, these genes have roles in generating courtship songs and represent interesting options for studying species complexes. In addition, the correlated evidence obtained from different approaches has been adequate in addressing the species complex question (Costa & Stanewsky 2013). Male copulation song data along with *per* gene polymorphisms (Souza et al. 2004, Vigoder et al. 2010), or with *para* gene variation (Lins et al. 2012), have resulted in even more robust evidence. Moreover, correlations between the distribution of allele frequencies of microsatellite *loci* and male aggregation-sex pheromones-types (Maingon et al. 2003, Watts et al. 2005), and *per* gene variation data combined with copulation song patterns (Vigoder et al. 2010), allowed the recognition of *Lu. cruzi* Mangabeira, 1938, as another sibling species within the *Lu. longipalpis* complex (Watts et al. 2005, Vigoder et al. 2010). In the same way, sandflies from Posadas (Misiones state, Argentina) might represent yet another sibling species, different from those found in the Northeast and Southeast regions of Brazil (Salomón et al. 2010).

An integrative analysis using a combination of biochemical, behavioral and molecular traits (Araki et al. 2009) strongly supports the hypothesis of two main groups within the *Lu. longipalpis* complex in Brazil. One group is a genetically homogeneous species whose males produce the Burst-type copulation song and the Cemb-1 pheromone (Cemb-1/Burst). The other group is genetically heterogeneous and probably represents a number of sibling species with different levels of divergence. Males of this latter group produce different subtypes of the Pulse-type copulation song (P1 to P5) in combination with different sex pheromones (9MGB, 9MGB^+^, 3MαH, Cemb-1 and Cemb-2). More recently, *para* gene variation was found in agreement with the two-group hypothesis (Lins et al. 2012). Moreover, this molecular marker showed diagnostic fixed polymorphisms, which can be used as a reliable indicator of two species. In addition, comparisons of life cycles between siblings species showed that populations from the second more heterogeneous group, such as from Jacobina (3MαH/P1), Lapinha (9MGB/P2) and Sobral 1S (9MGB^+^/P3), more easily adapt to the conditions of laboratory than do populations from Natal and Sobral 2S, which belong to the Cemb-1/Burst group. These phenological differences are a further indication of the differentiation between two main groups of the *Lu. longipalpis* species complex (Souza et al. 2009).

When studying a species complex, the existence of two putative species in sympatry is one of the strongest pieces of evidence that they are indeed distinct. In Brazil, this scenario has been observed in at least four localities, as mentioned previously (reviewed by Vigoder et al. 2015, Spiegel et al. 2016). At these localities, males can be distinguished by the number of abdominal pale spots, which is supported by molecular analysis, and so these two phenotypes are considered to be two sympatric species at Sobral (Bauzer et al. 2002b, Bottecchia et al. 2004, Watts et al. 2005, Lins et al. 2008, Araki et al. 2013), Estrela de Alagoas and Jaíba (Araki et al. 2009, Lins et al. 2012). It is expected that future molecular analysis with samples from Palmas and Porto Nacional will also show differentiation at the molecular level.

Incongruent evidence shown by some molecular markers (e.g., variable levels of divergence and phylogenetic relationships) could be due to different rates of evolution, introgression between counterparts, or the relative brief time of divergence among members of this species complex, and could explain the conflicting interpretations among early studies of Brazilian populations. For example, the *per* gene was considered a useful molecular marker in studies of population genetics, and even more so considering the additional evidence from pheromones and copulation song analysis (Bauzer et al. 2002a, b, Araki et al. 2009) and the fixed polymorphisms detected in nearby populations in Northeast Brazil (Lima-Costa Jr et al. 2015). The published *per* data were reanalysed along with sequences deposited in Genbank in 2004 by Meneses and collaborators (unpublished observations) using different phylogenetic methods and found low bootstrap support and numerous polytomies (Golczer & Arrivillaga 2010). These findings are compatible with rapidly evolving markers, and indicates multiple speciation events and, further, recombination and introgression (Araki et al. 2013). On the other hand, mitochondrial markers are very commonly used for systematics because of their slow evolutionary rate and low recombination, but they also present some restrictions. Some studies questioned the use of mtDNA alone to explore phylogenetic relationships between closely related taxa, especially in cases with introgression (Hurst & Jiggins 2005, Galtier et al. 2009). More recently developed barcode analysis does not seem to be suitable for species recognition in *Lu. longipalpis* species complex due to introgression, but is more promising for higher taxonomic levels (Pinto et al. 2015).

A multi-locus approach was undertaken to estimate and compare levels of divergence and gene flow for 21 nuclear loci (including *cac*, *para* and *per*) between the sympatric siblings from Sobral (1S: 9MGB^+^/P3 and 2S: Cemb-1/B) and two allopatric species from the localities of Lapinha (9MGB/P2) and Pancas (Cemb-1/B) in Southeast Brazil (Araki et al. 2013). The nuclear data fit the isolation with migration model of speciation and reveals that introgressive hybridisation has played a crucial role in speciation of the lineages Cemb-1/Burst and 9MGB/Pulse (P2 and P3), which occurred in allopatry at around 0.5 MYA (Fig. 3). Following secondary contact and another period of hybridisation, reinforcement of reproductive isolation might have promoted the evolution of more efficient mate discrimination, such as the recognition of conspecific male aggregation-sex pheromones and copulation songs, and/or other isolation mechanisms (Machado et al. 2007, Servedio 2004). Perhaps differences in life cycle traits (Souza et al. 2009) and patterns of locomotor activity (Rivas et al. 2008) are the results of divergence process of the two sympatric siblings.

**Fig. 3:**
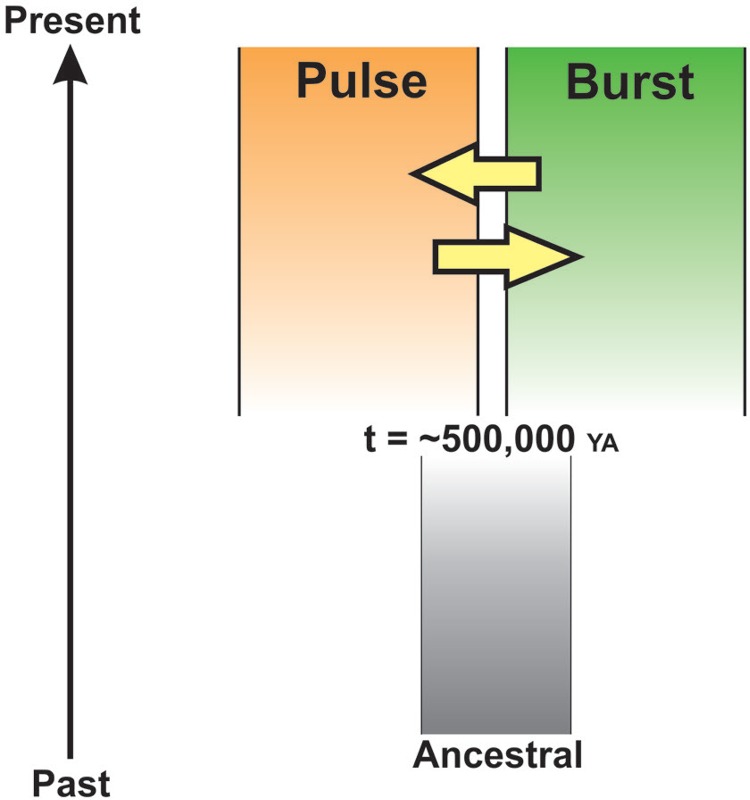
: the isolation with migration model of speciation. The graphic illustrates comparisons between sympatric and allopatric Brazilian populations of *Lutzomyia longipalpis* using 21 nuclear loci. An ancestral population separated into two descendant groups, Pulse and Burst, at approximately 500,000 YA. The yellow arrows represent migrations between counterparts.

*Epidemiology* - The sandfly *Lu. longipalpis s.l.* is the most important Neotropical vector of *Leishmania (Leishmania) infantum* Nicolle 1908, the causative agent of American visceral leishmaniasis (AVL). Formerly AVL was associated with rural and peri-urban areas, but more recently dispersion and urbanisation has been the most relevant epidemiological change observed in Brazil, Paraguay and Argentina (Salomón et al. 2015). In Brazil, AVL used to occur mainly in the Northeast region (Romero & Boelaert 2010), but has since spread to urban centers in the Central-West and Southeast regions. In the last three decades, the disease has begun to move into urban areas and the pattern observed suggests minor active dispersion activity by sandflies but a more significant passive component of dispersal, such as the transportation of soil from rural regions to cities (Brazil 2013a). In contrast, in Venezuela and Colombia, AVL still occurs mainly in rural areas, and no increases in the frequency of urban cases has been observed (Salomón et al. 2015).

Besides AVL, *L. infantum* causes atypical American cutaneous leishmaniasis (ACL) in Central and South America. This clinical pleomorphism might be due to sandfly genetic variability, as well as the genetic variability of *Leishmania* species, host susceptibility and immune status, and/or environmental factors. It is likely that *Leishmania* transmission, virulence and clinical outcome are influenced by coevolutionary interactions between specific *Leishmania* and specific sandfly genotypes (Maingon et al. 2008a). A comprehensive study of the population structure of *L. infantum* in the New World was carried out using several microsatellite *loci* and at least three main populations were identified (Kuhls et al. 2011, Ferreira et al. 2012). The existence of a link between these recently identified *Leishmania* groups and the species of the *Lu. longipalpis* species complex remains to be elucidated.

*Lu. longipalpis s.l*. is a highly anthropophilic species, and sick dogs and foxes, reservoirs of *L. infantum*, have often been found naturally infected (Deane 1956, Lainson & Shaw 1979, 1998, Ryan et al. 1984). These reports stimulated a great need to demonstrate transmission by the bite of *Lu. longipalpis* under experimental conditions. Although this sandfly has been shown to be more likely to establish colonies in the laboratory, the parasite-host relationship is still not fully elucidated, however, there is some evidence. In the early 1960’s, Sherlock and Sherlock (1961) were conducting studies on experimental infection of *Lu. longipalpis* from Fortaleza (CE) and reported variation in the ability of this sandfly to infect and transmit *L. infantum* in different areas of Brazil. The first successful experimental transmission was that of Lainson et al. (1977), who demonstrated the transmission of the parasite to a hamster through the bite of *Lu. longipalpis* from Morada Nova reared in laboratory, although nothing was mentioned about differential capabilities. In a more recent study, Warburg et al. (1994) suggested that components in the saliva of the vector may play a role in inducing the impairment of liver and spleen and not the parasite. Since *L. infantum* transmitted by sandflies usually causes AVL in Brazil and Colombia, while infections in Central America usually result in skin lesions, the authors claim that maxadilan is more potent in insects found in Brazil and Colombia than in Costa Rica. They were able to demonstrate that sandflies in Costa Rica are vectors of ACL because the parasites remain in the skin due to very low vasodilator activity with little effect from the maxadilan in their saliva, thus leading to the cutaneous form of the disease. The sandflies in Brazil and Colombia have a great amount of maxadilan, which exarcerbates even a minor skin infection, allowing the parasites to invade even the liver and spleen, leading to visceral leishmaniasis. These findings led the authors to suggest that *Lu. longipalpis* is a complex species that may modulate the pathology of the disease they transmit depending on the amount of maxadilan. On the other hand, a study by Maingon et al. (2008b) has led to speculation about the association between environmental factors and host response to vector-transmitted parasitic disease. In Honduras it has been reported that ACL and AVL are caused by apparently genetically identical *L. infantum* (Noyes et al. 1997), and that inorganic particles of volcanic origin accumulated in the salivary gland might have an immunomodulatory effect and alter the virulence of *Leishmania* (Maignon et al. 2008b). More recently, Casanova et al. (2006) reported that *Lu. longipalpis* from Araçatuba and Espírito Santo do Pinhal (SP, Brazil) produced different aggregation-sex pheromones, 9MGB and Cemb-1, respectively. This observation, coupled with the remarkable difference between the epidemiological frameworks, suggests an indirect and different vectorial capacity. It is worth emphasizing that experimental comparisons of infections by *Lu. longipalpis* of the two main pheromone/song types with *L. infantum* remains still a matter in need of special attention. In particular, such comparisons would be important in areas of sympatry such as Sobral, Estrela de Alagoas, Palmas and Porto Nacional.

*Concluding remarks* - Since its first description as *Ph. longipalpis* by Lutz and Neiva in 1912, the systematics of *Lu. longipalpis s.l.*, has undergone revisions with the continual acquisition of new knowledge. Presently, the existence of a *Lu. longipalpis* species complex is accepted and has raised the prospect of assigning valid taxonomic names to its included species (Brandão-Filho et al. 2009). Although a few morphologic studies have shown differences among some populations (de la Riva et al. 2001, Santos et al. 2015), no discrete anatomical attribute has proven to be reliably diagnostic and extensively employed. The exact number of sibling species in the *Lu. longipalpis* species complex remains unclear, but at least seven different species have been suggested in Brazil alone (Araki et al. 2009), and additional species certainly exist according to more recent data (Table, Fig. 4). To better understand the interesting radiation of this group, a research strategy of combining approaches will probably prove productive in demonstrating how many species are in the *Lu. longipalpis* complex and the relationships and divergences among them. The advancement of next generation sequencing technologies provides an opportunity to explore molecular variation on a larger scale, which may lead to better understand of the molecular evolution of this interesting group. Further analysis throughout the genome is needed to better understand whether loci related to vectorial capacity can influence the transmission dynamics of *Leishmania* parasites by the different *Lu. longipalpis* sibling species. Furthermore, it would be interesting to investigate whether a particular population of *Leishmania* can be correlated with different species of this complex, as well as possible relationships with clinical pleomorphism.

**Fig. 4:**
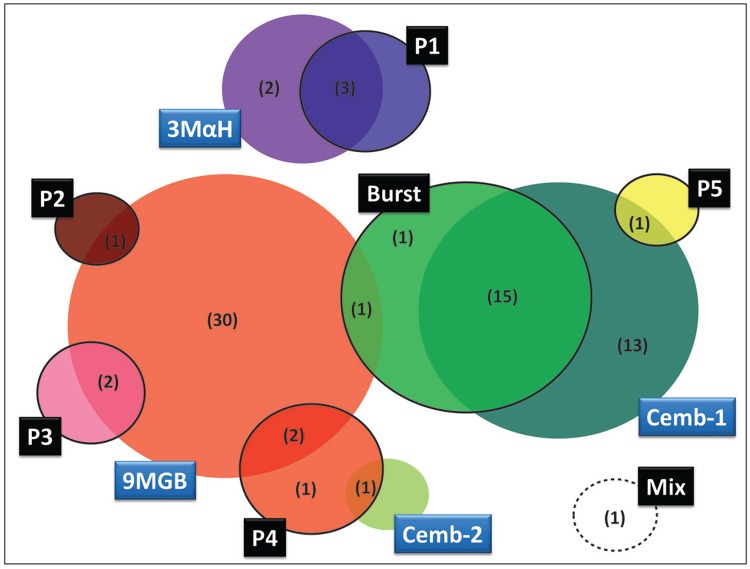
: diagram summarising the available pheromones and copulation songs data. Differential male pheromones (blue light box): Cembrene-1 and Cembrene-2 (Cemb-1 and Cemb-2, respectively), (*S*)-9-methyl-germacrene-B (9MGB) and (*1S,3S,7R*)-3-methyl-α-himachalene (3MαH). Types and sub-types of male pheromones (black box): Burst, Pulse (subtypes P1 to P5) and Mix. The number of populations analysed is shown in brackets.

The knowledge of chemical communication in *Lu. longipalpis s.l.* has advanced remarkably (reviewed by Spiegel et al. 2016), and is contributing to an alternative strategy for the control of this sandfly (Brazil 2013b). The use of synthetic (S)-9-methylgermacrene-B and the analogue (+/-)-9-methylgermacrene have shown to be useful in disrupting mating because females are highly attracted to these compounds (Hamilton 2008, Bray et al. 2010). Moreover, the attractiveness of the synthetic sex pheromones to males avoids the formation of lek aggregations, which would be helpful for sandfly population management (Vanessa Barbosa, personal communication). The use of this approach represents an interesting alternative strategy for vector control programs. Insecticide resistance of *Lu. longipalpis s.l.* has not yet been fully studied, however, there are some indications of its occurrence (Coutinho-Abreu et al. 2007, Alexander et al. 2009). The differential and reduced susceptibilities assessed among sandflies from the localities of Lapinha and Morada Nova (Alexander et al. 2009) indicate the need to take into consideration the pattern of insecticide resistance among siblings species of *Lu. longipalpis s.l.* in control strategies in Brazil and in other countries endemic for AVL.

The wide variety of evidence, including chemical, behavioral and molecular traits, suggests very recent speciation and complex population structure in the *Lu. longipalpis* species complex. Extending studies to other populations will give us a better sense of the geographical distribution of the sibling species of *Lu. longipalpis* and clarify their particularities, especially relative to their potential implication in incidence of AVL. Although significant advances have been achieved to date, differential vectorial capacity and the correlation between genetic structure of parasite and vectors populations remain to be elucidated. Furthermore, increased knowledge regarding recent epidemiological changes, such as urbanisation, is essential for pursuing effective strategies for sandfly control in the New World.

## References

[B1] Agassiz L (1846). Nomenclatoris zoologici. Index universalis: continens nomina systematica
classium, ordinum, familiarum et generum animalium omnium, tam viventium quam
fossilium: secundum ordinem alphabeticum unicum disposita, adjectis homonymiis
plantarum.

[B2] Alexander B, Barros VC, Souza SS, Teodoro LP, Soares ZR, Gontijo NF (2009). Susceptibility to chemical insecticides of two Brazilian populations
of the visceral leishmaniasis vector Lutzomyia longipalpis
(Diptera:Psychodidae). Trop Med Inter Health.

[B3] Araki AS, Ferreira GE, Mazzoni CJ, Souza NA, Machado RC, Bruno RV (2013). Multilocus analysis of divergence and introgression in sympatric and
allopatric sibling species of the Lutzomyia longipalpis complex in
Brazil. PLoS Negl Trop Dis.

[B4] Araki AS, Vigoder FM, Bauzer LG, Ferreira GE, Souza NA, Araújo IB (2009). Molecular and behavioral differentiation among Brazilian populations
of Lutzomyia longipalpis (Diptera: Psychodidae: Phlebotominae). PLoS Negl Trop Dis.

[B5] Arrivilaga J, Feliciangeli MD (2001). Lutzomyia pseudolongipalpis: the first new species within the
longipalpis (Diptera: Psychodidae: Phlebotominae) complex from La Rinconada,
Curarigua, Lara state, Venezuela. J Med Entomol.

[B6] Arrivillaga J, Mutebi JP, Piñango H, Norris D, Alexander B, Feliciangeli MD (2003). The taxonomic status of genetically divergent populations of Lutzomyia
longipalpis (Diptera: Psychodidae) based on the distribution of mitochondrial and
isozyme variation. J Med Entomol.

[B7] Arrivillaga JC, Norris DE, Feliciangeli MD, Lanzaro GC (2002). Phylogeography of the neotropical sandfly Lutzomyia longipalpis
inferred from mitochondrial DNA sequences. Infect Genet Evol.

[B8] Arrivillaga JC, Rangel Y, Oviedo M, Feliciangeli MD (2000). Genetic divergence among Venezuelan populations of Lutzomyia
longipalpis (Diptera: Psychodidae: Phlebotominae). J Med Entomol.

[B9] Balbino VQ, Coutinho-Abreu IV, Sonoda IV, Melo MA, Andrade PP, Castro JA (2006). Genetic structure of natural populations of the sandfly Lutzomyia
longipalpis (Diptera: Psychodidae) from the Brazilian northeastern
region. Acta Trop.

[B10] Barretto MP (1962). Novos subgêneros de Lutzomyia França, 1924 (Diptera, Psychodidae,
subfamília Phlebotominae). Rev Inst Med Trop São Paulo.

[B11] Bauzer LG, Gesto JS, Souza NA, Ward RD, Hamilton JG, Kyriacou CP (2002a). Molecular divergence in the period gene between two putative sympatric
species of the Lutzomyia longipalpis complex. Mol Biol Evol.

[B12] Bauzer LG, Souza NA, Ward RD, Kyriacou CP, Peixoto AA (2002b). The period gene and genetic differentiation between three Brazilian
populations of Lutzomyia longipalpis. Insect Mol Biol.

[B13] Bauzer LGSR, Souza NA, Maingon RDC, Peixoto AA (2007). Lutzomyia longipalpis in Brazil: a complex or a single species? A
mini-review. Mem Inst Oswaldo Cruz.

[B14] Bonnefoy S, Tibayrenc M, Le Pont F, Dujardin J, Desjeux P, Ayala F (1986). An isozymic study of Lutzomyia longipalpis (Diptera: Psychodidae) the
vector of visceral leishmaniasis in the Yungas (Bolivia). Cah ORSTOM Ser Entomol Med Parasitol.

[B15] Bottecchia M, Oliveira SG, Bauzer LG, Souza NA, Ward RD, Garner KJ (2004). Genetic divergence in the cacophony IVS6 intron among five Brazilian
populations of Lutzomyia longipalpis. J Mol Evol.

[B16] Brandão SP, Balbino VQ, Marcondes CB, Brazil RP, Hamilton JG, Shaw JJ (2009). Should reproductively isolated populations of Lutzomyia longipalpis
sensu lato receive taxonomically valid names?. Mem Inst Oswaldo Cruz.

[B17] Bray DP, Alves GB, Dorval ME, Brazil RP, Hamilton JGC (2010). Synthetic sex pheromone attracts the leishmaniasis vector Lutzomyia
longipalpis to experimental chicken sheds trated with insecticide. Parasit Vectors.

[B18] Bray DP, Hamilton GC (2007). Courtship behavior in the sandfly Lutzomyia longipalpis, the New World
vector of visceral leishmaniasis. Med Vet Entomol.

[B19] Brazil RP, Caballero NN, Hamilton JG (2009). Identification of the sex pheromone of Lutzomyia longipalpis (Lutz
& Neiva, 1912) (Diptera: Psychodidae) from Asunción, Paraguay. Parasit Vectors.

[B20] Brazil RP, Andrade W, Santos A, Parente J, Hamilton J (2010). Presença de dois morfotipos de Lutzomyia longipalpis (Diptera: Psychodidae)
ocorrendo em simpatria em Porto Nacional, estado do Tocantins.

[B21] Brazil RP, Hamilton JGCH (2002). Isolation and identification of 9-methylgermacrene-B as the putative
sex pheromone of Lutzomyia cruzi (Mangabeira, 1938) (Diptera:
Psychodidae). Mem Inst Oswaldo Cruz.

[B22] Brazil RP (2013a). The dispersion of Lutzomyia longipalpis in urban areas. Rev Soc Bras Med Trop.

[B23] Brazil RP (2013b). The use of sex pheromone in the control of the Lutzomyia longipalpis
(Psychodidae: Phlebotominae), the vector of Leishmania infantum in the New
World. Entomol Ornithol Herpetol.

[B24] Casanova C, Colla-Jacques FE, Hamilton JG, Brazil RP, Shaw JJ (2015). Distribution of Lutzomyia longipalpis chemotype populations in São
Paulo state, Brazil. PLoS Negl Trop Dis.

[B25] Casanova C, Hamilton JGC, Trigo JR, Costa AIP (2006). Identification of sex pheromones of Lutzomyia longipalpis (Lutz &
Neiva, 1912) populations from the state of São Paulo, Brazil. Mem Inst Oswaldo Cruz.

[B26] Coquillet DW (1907). Discovery of blood sucking Psychodidae in America. Entomological News.

[B27] Costa R, Stanewsky R (2013). When population and evolutionary genetics met
behaviour. Mem Inst Oswaldo Cruz.

[B28] Coutinho-Abreu IV, Balbino VQ, Valenzuela JG, Sonoda IV, Ramalho-Ortigão JM (2007). Structural characterization of acetylcholinesterase 1 from the sand
fly Lutzomyia longipalpis (Diptera: Psychodidae). J Med Entomol.

[B29] Coutinho-Abreu IV, Sonoda IV, Fonseca JA, Melo MA, Balbino VQ, Ramalho-Ortigão M (2008). Lutzomyia longipalpis s.l. in Brazil and the impacto of the São
Francisco River in the speciation of this sand fly vector. Parasit Vectors.

[B30] Azevedo ACR, Monteiro FA, Cabello PH, Souza NA, Rosa-Freitas MG, Rangel EF (2000). Studies on populations of Lutzomyia longipalpis (Lutz & Neiva,
1912) (Diptera: Psychodidae: Phlebotominae) in Brazil. Mem Inst Oswaldo Cruz.

[B31] Riva J, Le Pont F, Ali V, Matias A, Mollinedo S, Dujardin JP (2001). Wing geometry as a tool for studying the Lutzomyia longipalpis
(Diptera: Psychodidae) complex. Mem Inst Oswaldo Cruz.

[B32] Deane LM (1956). Leishmaniose visceral no Brasil. Estudos sobre reservatórios e transmissores
realizados no estado do Ceará.

[B33] Dujardin JP, Torrez EM, Le Pont F, Hervas D, Sossa D (1997). Isozymic and metric variation in the Lutzomyia longipalpis
complex. Med Vet Entomol.

[B34] Dyar HG, Nuñez-Tovar M (1926). Notes on biting flies from Venezuela. Insec Inscit Menst.

[B35] Fairchild GB, Hertig M (1958). Notes on the Phlebotomus of Panama XV four apparently new
synonymies. Proc Ent Soc Washinton.

[B36] Ferreira GEM, Santos BN, Dorval MEC, Ramos TPB, Porrozi R, Peixoto AA (2012). The genetic structure of Leishmania infantum populations in Brazil and
its possible association with the transmission cycle of visceral
leishmaniasis. PLoS ONE.

[B37] França C (1920). Observations sur le genre Phlebotomus. II. Phlebotomes du Nouveau
Monde (Phlebotomus du Brésil et du Paraguay). Bull Soc Port Sci Nat.

[B38] Galati EAB, Rangel EF, Lainson R (2003). Morfologia e taxonomia: classificação de
Phlebotominae. Flebotomíneos do Brasil.

[B39] Galati EAB (1995). Philogenetic systematics of the Phlebotominae (Diptera, Psychodidae)
with emphasis on American groups. Bol Mal Salud Amb.

[B40] Galliard H (1934). Um Phlebotome nouveau de Yucatan, Phlebotomus almazani n.
sp.. Ann Parasit Hum Comp.

[B41] Galtier N, Nabholz S, Glémin, Hurst DD (2009). Mitochondrial DNA as a marker of molecular diversity: a
reappraisal. Mol Ecol.

[B42] Golczer G, Arrivillaga J (2010). Gen periodo no construye filogenias dentro del complejo de especie,
Lutzomyia longipalpis (Diptera: Phlebotominae). Metodos en Ecología y Sistemática.

[B43] Hamilton JG, Brazil RP, Maingon R (2004). A fourth chemotype of Lutzomyia longipalpis (Diptera: Psychodidae)
from Jaiba, Minas Gerais state, Brazil. J Med Entomol.

[B44] Hamilton JG, Dougherty MJ, Ward RD (1994). Sex pheromone activity in a single component of tergal gland extract
of Lutzomyia longipalpis (Diptera: Psychodidae) from Jacobina. J Chem Ecol.

[B45] Hamilton JG, Maingon RD, Alexander B, Ward RD, Brazil RP (2005). Analysis of the sex pheromone extracts of individual male Lutzomyia
longipalpis sandflies from six regions in Brazil. Med Vet Entomol.

[B46] Hamilton JG, Ward RD, Dougherty MJ, Maignon R, Ponce C, Ponce E (1996c). Comparison of the sex-pheromone components of Lutzomyia longipalpis
(Diptera: Psychodidae) from areas of visceral and atypical cutaneous leishmaniasis
in Honduras and Costa Rica. Ann Trop Med Parasitol.

[B47] Hamilton JG, Ward RD (1991). Gas-chromatographic analysis of Lutzomyia longipalpis tergal pheromone
gland extract. Parassitologia.

[B48] Hamilton JG (2008). Sandfly pheromones. Their biology and potential for use in control
programs. Parasite.

[B49] Hamilton JGC, Dawson GW, Pickett JA (1996a). 9-Methyl-germacrene B, a novel homosesquiterpene from sex pheromone
glands of Lutzomyia longipalpis (Diptera: Psychodidae) from Lapinha,
Brazil. J Chem Ecol.

[B50] Hamilton JGC, Hooper AM, Mori K, Pickett JA, Sano S (1996b). 3-Methyl-α himachalene is confirmed, and the relative stereochemistry
defined, by synthesis as the sex pheromone of the sandfly Lutzomyia longipalpis
from Jacobina, Brazil. Chem Commun.

[B51] Hemming F (1958). Official list of rejected and invalid generic names in Zoology.

[B52] Hodgkinson VH, Birungi J, Quintana M, Dietze R, Munstermann LE (2003). Mitochondrial cytochrome b variation in populations of the visceral
leishmaniasis vector Lutzomyia longipalpis across eastern Brazil. Am J Trop Med Hyg.

[B53] Hoikkala A, Crossley S, Castillo-Melendez C (2000). Copulatory courtship in Drosophila birchii and D. serrata, species
recognition and sexual selection. J Insect Behav.

[B54] Hoikkala A, Crossley S (2000). Copulatory courtship in Drosophila: behavior and songs of D. birchii
and D. serrata. J Insect Behav.

[B55] Hurst GD, Jiggins FM (2005). Problems with mitochondrial DNA as a marker in population,
phylogeographic and phylogenetic studies: the effects of inherited
symbionts. Proc Biol Sci.

[B56] Johansson BG, Jones TM (2007). The role of chemical communication in mate choice. Biol Rev Camb Philos Soc.

[B57] Kuhls K, Alam MZ, Cupolillo E, Ferreira GE, Mauricio IL, Oddone R (2011). Comparative microsatellite typing of new world leishmania infantum
reveals low heterogeneity among populations and its recent old world
origin. PLoS Negl Trop Dis.

[B58] Lainson R, Shaw JJ, Collier L, Balows A, Sussman M (1998). New World Leishmaniasis. The neotropical Leishmania
species. Topley & Wilson’s microbiology and microbiol infectious diseases.

[B59] Lainson R, Shaw JJ, Lumsden WHR, Evans DA (1979). The role of animals in the epidemiology of South
American Leishmaniasis. Biology of the Kinetoplastida.

[B60] Lainson R, Ward RD, Shaw JJ (1977). Experimental transmission of Leishmania chagasi, causative agent of
neotropical visceral leishmaniasis, by the sandfly Lutzomyia
longipalpis. Nature.

[B61] Lampo M, Torgerson D, Márquez LM, Rinaldi M, García CZ, Arab A (1999). Occurrence of sibling species of Lutzomyia longipalpis (Diptera:
Psychodidae) in Venezuela: first evidence from reproductively isolated sympatric
populations. Am J Trop Med Hyg.

[B62] Lane RP, Phillips A, Molyneux DH, Procter C, Ward RD (1985). Chemical analysis of the abdominal glands of two forms of the
Lutzomyia longipalpis: site of a possible sex pheromone?. Ann Trop Med Parasit.

[B63] Lane RP, Ward RD (1984). The morphology and possible function of abdominal patches in males of
two forms of the leishmaniasis vector Lutzomyia longipalpis (Diptera:
Phlebotominae). Cah ORSTOM Ser Ent Med Parasitol.

[B64] Lanzaro GC, Alexander B, Mutebi J-P, Montoya-Lerma J, Warburg A (1998). Genetic variation among natural and laboratory colony populations of
Lutzomyia longipalpis (Lutz & Neiva, 1912) (Diptera: Psychodidae) from
Colombia. Mem Inst Oswaldo Cruz.

[B65] Lanzaro GC, Lopes AH, Ribeiro JM, Shoemaker CB, Warburg A, Soares M (1999). Variation in the salivary peptide, maxadilan, from species in the
Lutzomyia longipalpis complex. Insect Mol Biol.

[B66] Lanzaro GC, Ostrovska K, Herrero MV, Lawyer PG, Warburg A (1993). Lutzomyia longipalpis is a species complex: genetic divergence and
interspecific hybrid sterility among three populations. Am J Trop Med Hyg.

[B67] Lima-Costa CR, Freitas MT, Figueredo CAS, Aragão NC, Silva LG, Marcondes CB (2015). Genetic structuring and fixed polymorphisms in the period among
natural populations of Lutzomyia longipalpis in Brazil. Parasit Vectors.

[B68] Lins RM, Souza NA, Brazil RP, Maingon RD, Peixoto AA (2012). Fixed differences in the paralytic gene define two lineages within the
Lutzomyia longipalpis complex producing different types of courtship
songs. PLoS ONE.

[B69] Lins RMMA, Souza NA, Peixoto AA (2008). Genetic divergence between two sympatric species of the Lutzomyia
longipalpis complex in the paralytic gene, a locus associated with insecticide
resistance and lovesong production. Mem Inst Oswaldo Cruz.

[B70] Lutz A, Neiva A (1912). Contribuição para o conhecimento das espécies do gênero Phlebotomus
existentes no Brasil. Mem Inst Oswaldo Cruz.

[B71] Machado CA, Haselkorn TS, Noor MA (2007). Evaluation of the genomic extent of effects of fixed inversion
differences on intraspecific variation and interspecific gene flow in Drosophila
pseudoobscura and D. persimilis. Genetics.

[B72] Maingon RD, Khela A, Sampson C, Ward R, Walker K, Exley C (2008b). Aluminium: a natural adjuvant in Leishmania transmission via sand
flies?. Trans R Soc Trop Med Hyg.

[B73] Maingon RD, Ward RD, Hamilton JG, Bauzer LG, Peixoto AA (2008a). The Lutzomyia longipalpis species complex: does population
sub-structure matter to Leishmania transmission?. Trends Parasitol.

[B74] Maingon RD, Ward RD, Hamilton JG, Noyes HA, Souza N, Kemp SJ (2003). Genetic identification of two sibling species of Lutzomyia longipalpis
(Diptera: Psychodidae) that produce distinct male sex pheromones in Sobral, Ceará
state, Brazil. Mol Ecol.

[B75] Mangabeira O (1969). Sobre a sistemática e biologia dos Phlebotomus do
Ceará. Rev Bras Malariol Doencas Trop.

[B76] Morrison CA, Munstermann LE, Ferro C, Pardo R, Torres M (1995). Ecological and genetic studies of Lutzomyia longipalpis in a central
Colombian focus of visceral leishmaniasis. Bol Dir Malariol San Amb.

[B77] Morton IE, Ward RD (1989). Laboratory response of female Lutzomyia longipalpis sandflies to a
host and male pheromone source over distance. Med Vet Entomol.

[B78] Mukhopadhyay J, Ghosh K, Azevedo AC, Rangel EF, Munstermann LE (1998a). Genetic polymorphism of morphological and biochemical characters in a
Natal, Brazil, population of Lutzomyia longipalpis (Diptera:
Psychodidae). J Am Mosq Control Assoc.

[B79] Mukhopadhyay J, Ghosh K, Rangel EF, Munstermann LE (1998b). Genetic variability in biochemical characters of Brazilian field
populations of the Leishmania vector, Lutzomyia longipalpis (Diptera:
Psychodidae). Am J Trop Med Hyg.

[B80] Mukhopadhyay J, Rangel EF, Ghosh K, Munstermann LE (1997). Patterns of genetic variability in colonized strains of Lutzomyia
longipalpis (Diptera: Psychodidae) and its consequences. Am J Trop Med Hyg.

[B81] Mutebi JP, Alexander B, Sherlock I, Wellington J, Souza AA, Shaw J (1999). Breeding structure of the sandfly Lutzomyia longipalpis (Lutz &
Neiva) in Brazil. Am J Trop Med Hyg.

[B82] Mutebi JP, Rowton E, Herrero MV, Ponce C, Belli A, Valle S (1998). Genetic variability among populations of the sand fly Lutzomyia
(Lutzomyia) longipalpis (Diptera: Psychodidae) from Central
America. J Med Entomol.

[B83] Mutebi JP, Tripet F, Alexander JB, Lanzaro GC (2002). Genetic differentiation among populations of Lutzomyia longipalpis
(Diptera: Psychodidae) in Central and South America. Ann Entomol Soc Am.

[B84] Newman E (1834). Attempted division of British insects into natural
orders. Entomological Magazine.

[B85] Noyes H, Chance M, Ponce C, Ponce E, Maingon R (1977). Leishmania chagasi: genotypically similar parasites from Honduras
cause both visceral and cutaneous leishmaniasis in humans. Exp Parsitol.

[B86] Nuñez-Tovar M (1924). Mosquitos y flebótomos.

[B87] Oliveira SG, Bottecchia M, Bauzer LGSR, Souza NA, Ward RD, Kyriacou CP (2001). Courtship song genes and speciation in sand flies. Mem Inst Oswaldo Cruz.

[B88] Phillips A, Ward R, Ryan L (1986). Chemical analysis of compounds extracted from the tergal “spots” of
“Lutzomyia longipalpis” from Brazil. Acta Trop.

[B89] Pinto IS, das Chagas BD, Rodrigues AAF, Ferreira AL, Rezende HR, Bruno RV (2015). DNA barcoding of neotropical sand flies (Diptera, Psychodidae,
Phlebotominae): species identification and discovery within Brazil. PLoS ONE.

[B90] Rivas GB, Souza NA, Peixoto AA (2008). Analysis of the activity patterns of two sympatric sandfly siblings of
the Lutzomyia longipalpis species complex from Brazil. Med Vet Entomol.

[B91] Romero GA, Boelaert M (2010). Control of visceral leishmaniasis in Latin America - A systematic
review. PLoS Negl Trop Dis.

[B92] Rondani C (1840). Sopra una specie di insetto dittero. Memoria Prima per Servire alla Ditterologia Italiana.

[B93] Ryan L, Silveira FT, Lainson R, Shaw JJ (1984). Leishmanial infections in Lutzomyia longipalpis and Lu. antunesi
(Diptera:Psychodidae) on Island of Marajó, Pará state, Brazil. Trans R Soc Trop Med Hyg.

[B94] Salomón OD, Araki AS, Hamilton JGC, Acardi SA, Peixoto AA (2010). Sex pheromone and period gene characterization of Lutzomyia
longipalpis sensu lato (Lutz & Neiva) (Diptera: Psychodidae) from Posadas,
Argentina. Mem Inst Oswaldo Cruz.

[B95] Salomón OD, Feliciangeli MD, Quintana MG, Afonso MMS, Rangel EF (2015). Lutzomyia longipalpis urbanisation and control. Mem Inst Oswaldo Cruz.

[B96] Santos MFC, Andrade JD, Fernandes CES, Mateus NLF, Eguchi GU, Fernandes WD (2015). Morphometric analysis of Longipalpis (Diptera: Psychodidade) complex
populations in Mato Grosso do Sul, Brazil. J Med Entomol.

[B97] Santos MFC, Ribolla PEM, Alonso DP, Andrade JD, Casaril AE, Ferreira AMT (2013). Genetic structure of Lutzomyia longipalpis populations in Mato Grosso
do Sul, Brazil, based on microsatellite markers. PLos ONE.

[B98] Scopoli GA (1786). Deliciae florae et faunae insubricae, seu novae, aut minus cognitae
species plantarum et animalium, quas in insubria Austriaca tam spontanaes, quam
exoticas vidit, descripsit, et aeri incidi curavit. Ticini.

[B99] Servedio MR (2004). The what and why of research on reinforcement. PLoS Biol.

[B100] Sherlock IA, Sherlock VA (1961). On the experimental infection of “Phlebotomus longipalpis” by
“Leishmania donovani”. Rev Bras Biol.

[B101] Soto SI, Lehmann T, Rowton ED, Vélez BID, Porter CH (2001). Speciation and population structure in the morphospecies Lutzomyia
longipalpis (Lutz & Neiva) as derived from the mitochondrial ND4
gene. Mol Phylogenet Evol.

[B102] Souza NA, Andrade-Coelho CA, Silva VC, Ward RD, Peixoto AA (2009). Life cycle differences among Brazilian sandflies of the Lutzomyia
longipalpis sibling species complex. Med Vet Entomol.

[B103] Souza NA, Andrade-Coelho CA, Vigoder FM, Ward RD, Peixoto AA (2008). Reproductive isolation between sympatric and allopatric Brazilian
populations of Lutzomyia longipalpis s.l. (Diptera: Psychodidae). Mem Inst Oswaldo Cruz.

[B104] Souza NA, Vigoder FM, Araki AS, Ward RD, Kyriacou CP, Peixoto AA (2004). Analysis of the copulatory courtship songs of Lutzomyia longipalpis in
six populations from Brazil. J Med Entomol.

[B105] Souza NA, Ward RD, Hamilton JGC, Kyriacou CP, Peixoto AA (2002). Copulation songs in three siblings of Lutzomyia longipalpis (Diptera:
Psychodidae). Trans R Soc Trop Med Hyg.

[B106] Spiegel CN, Brazil RP, Soares MJ (2002). Ultrastructure of male sex pheromone glands in abdominal tergites of
five Lutzomyia sandfly species (Diptera: Psychodidae). Arthropod Struct Dev.

[B107] Spiegel CN, Dias DBS, Araki AS, Hamilton JGC, Brazil RP, Jones TM (2016). The Lutzomyia longipalpis complex: a brief natural history of
aggregation-sex pheromone communication. Parasit Vectors.

[B108] Steiger S, Stökl J (2014). The role of sexual selection in the evolution of chemical signals in
insects. Insects.

[B109] Theodor O (1948). Classification of the Old World species of the subfamily Phlebotominae
(Diptera: Psychodidae). Bull Entomol Res.

[B110] Uribe S (1999). The status of the Lutzomyia longipalpis species complex and possible
implications for Leishmania transmission. Mem Inst Oswaldo Cruz.

[B111] Vigoder FM, Araki AS, Bauzer LG, Souza NA, Brazil RP, Peixoto AA (2010). Lovesongs and period gene polymorphisms indicate Lutzomyia cruzi
(Mangabeira, 1938) as a sibling species of the Lutzomyia longipalpis (Lutz and
Neiva, 1912) complex. Infect Genet Evol.

[B112] Vigoder FM, Ritchie MG, Gibson G, Peixoto AA (2013). Acoustic communication in insect disease vectors. Mem Inst Oswaldo Cruz.

[B113] Vigoder FM, Souza NA, Brazil RP, Bruno RV, Costa LP, Ritchie MG (2015). Phenotypic differentiation in love song traits among sibling species
of the Lutzomyia longipalpis complex in Brazil. Parasit Vectors.

[B114] Warburg A, Saraiva E, Lanzaro GC, Titus RG, Neva F (1994). Saliva of Lutzomyia longipalpis sibling species differs in its
composition and capacity to enhance leishmaniasis. Philos Trans R Soc Lond B Biol Sci.

[B115] Ward RD, Phillips A, Burnet B, Marcondes CB, Service MW (1988). The Lutzomyia longipalpis complex: reproduction and
distribution. Biosystematics of haematophagous insects.

[B116] Ward RD, Ribeiro AL, Ready PD, Murtagh A (1983). Reproductive isolation between different forms of Lutzomyia
longipalpis (Lutz & Neiva) (Diptera: Psychodidae), the vector of Leishmania
donovani chagasi Cunha & Chagas, and its significance to kala-azar
distribution in South America. Mem Inst Oswaldo Cruz.

[B117] Ward RD, Ribeiro AL, Ryan L, Falcão AL, Rangel EF (1985). The distribution of two morphological forms of Lutzomyia longipalpis
(Lutz & Neiva) (Diptera: Psychodidae). Mem Inst Oswaldo Cruz.

[B118] Watts PC, Hamilton JG, Ward RD, Noyes HA, Souza NA, Kemp SJ (2005). Male sex pheromones and the phylogeographic structure of the Lutzomyia
longipalpis species complex (Diptera: Psychodidae) from Brazil and
Venezuela. Am J Trop Med Hyg.

[B119] Yin H, Mutebi JP, Marriott S, Lanzaro GC (1999). Metaphase karyotypes and G-banding in sandflies of the Lutzomyia
longipalpis complex. Med Vet Entomol.

[B120] Yin H, Norris DE, Lanzaro GC (2000). Sibling species in the Lutzomyia longipalpis complex differ in levels
of mRNA expression for the salivary peptide, maxadilan. Insect Mol Biol.

[B121] Young DG, Duncan MA (1994). Guide to the identification and geographic distribution of Lutzomyia
sand flies in Mexico, the West Indies, Central and South America (Diptera:
Psychodidae). Mem Amer Ent Inst.

